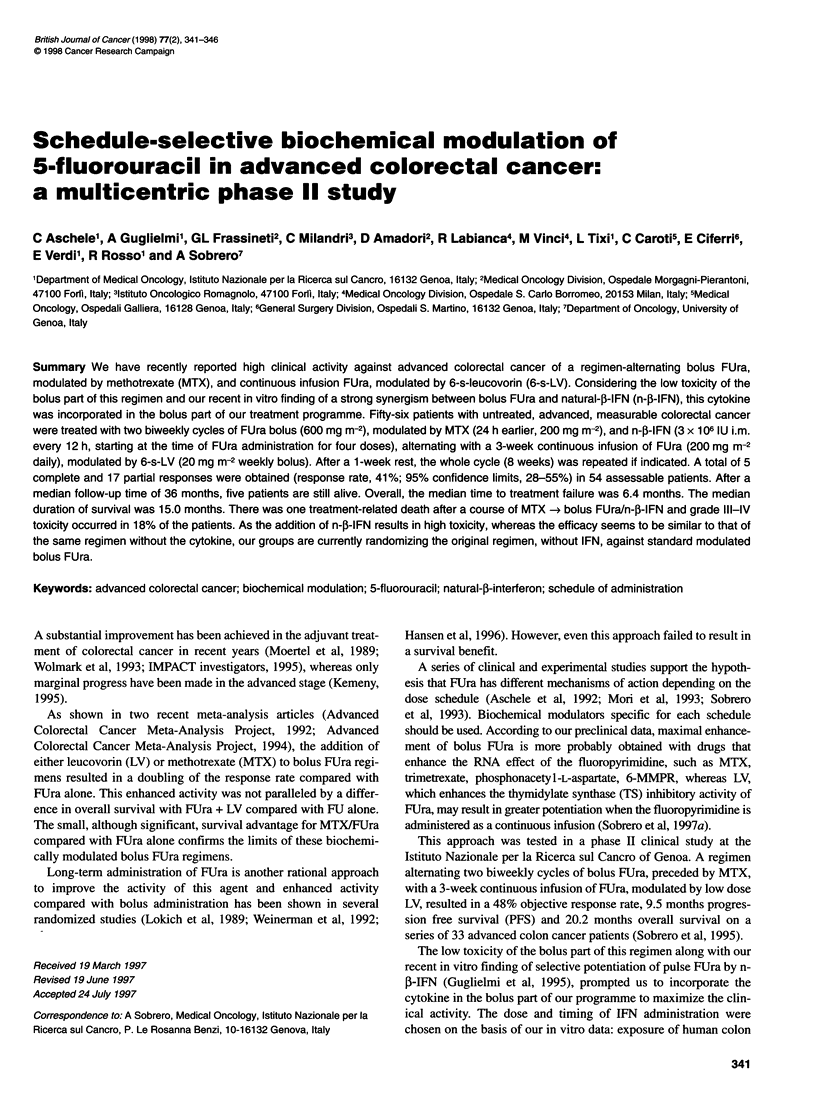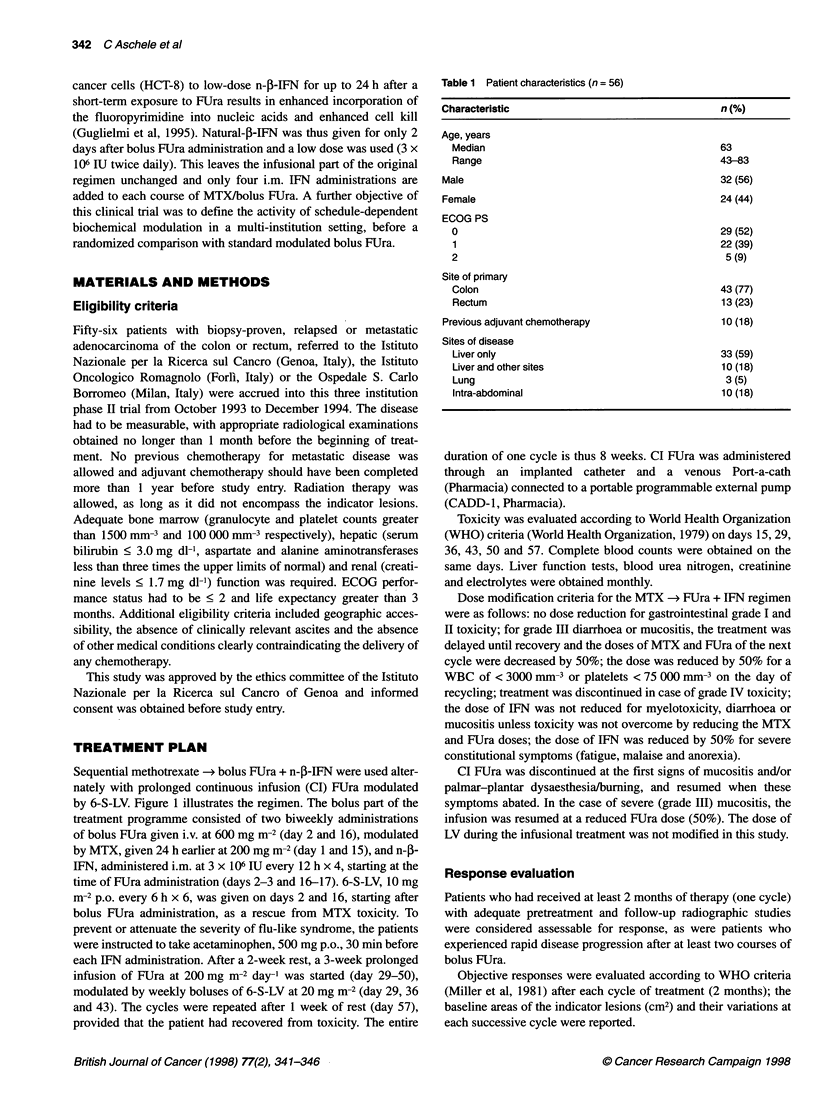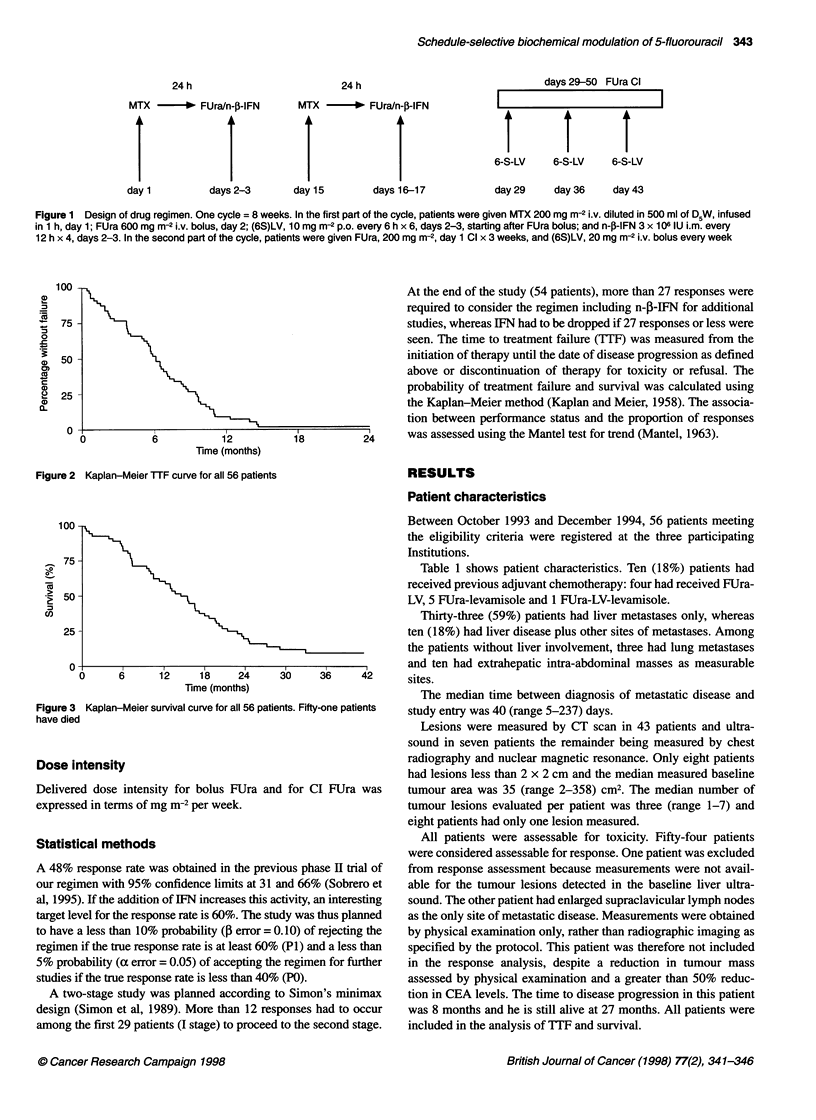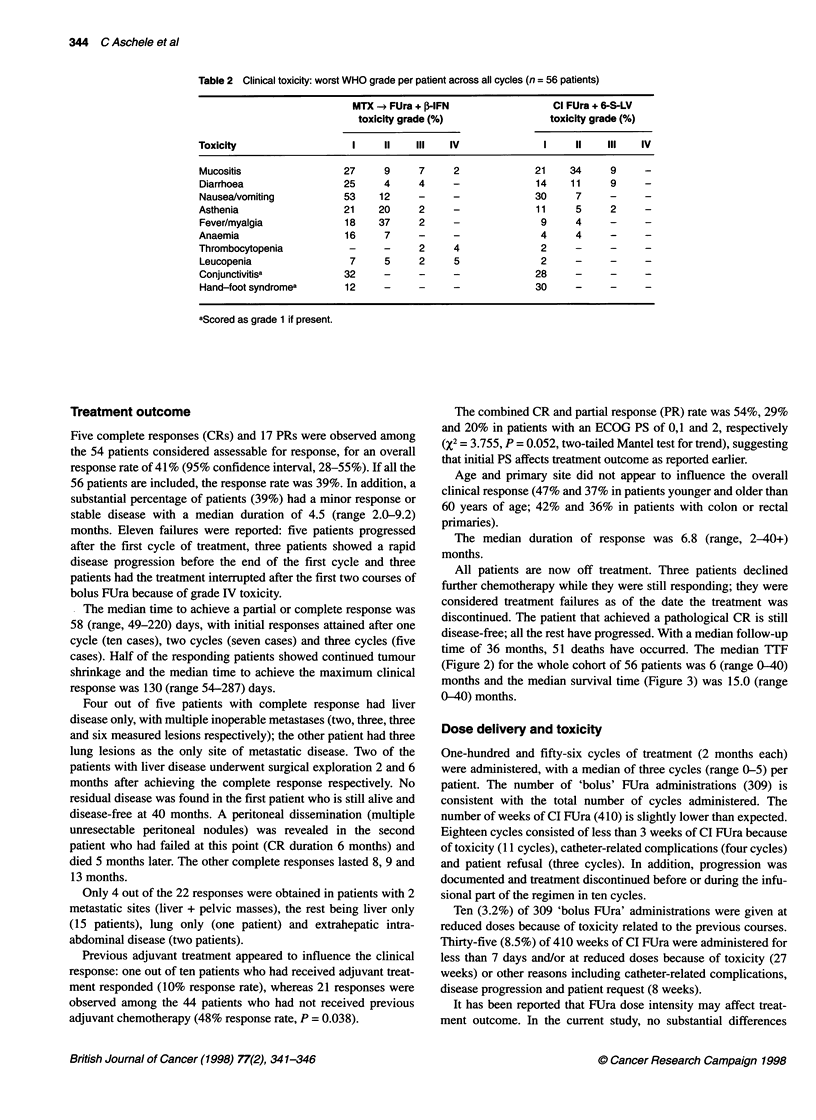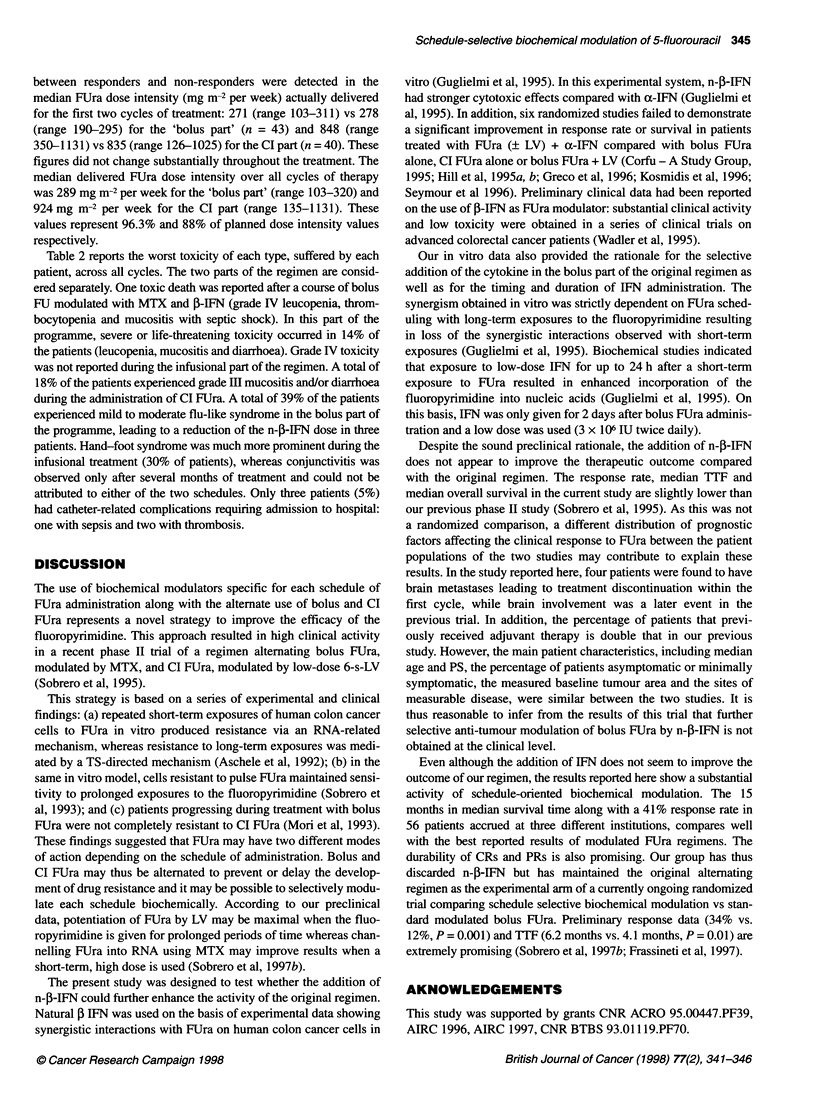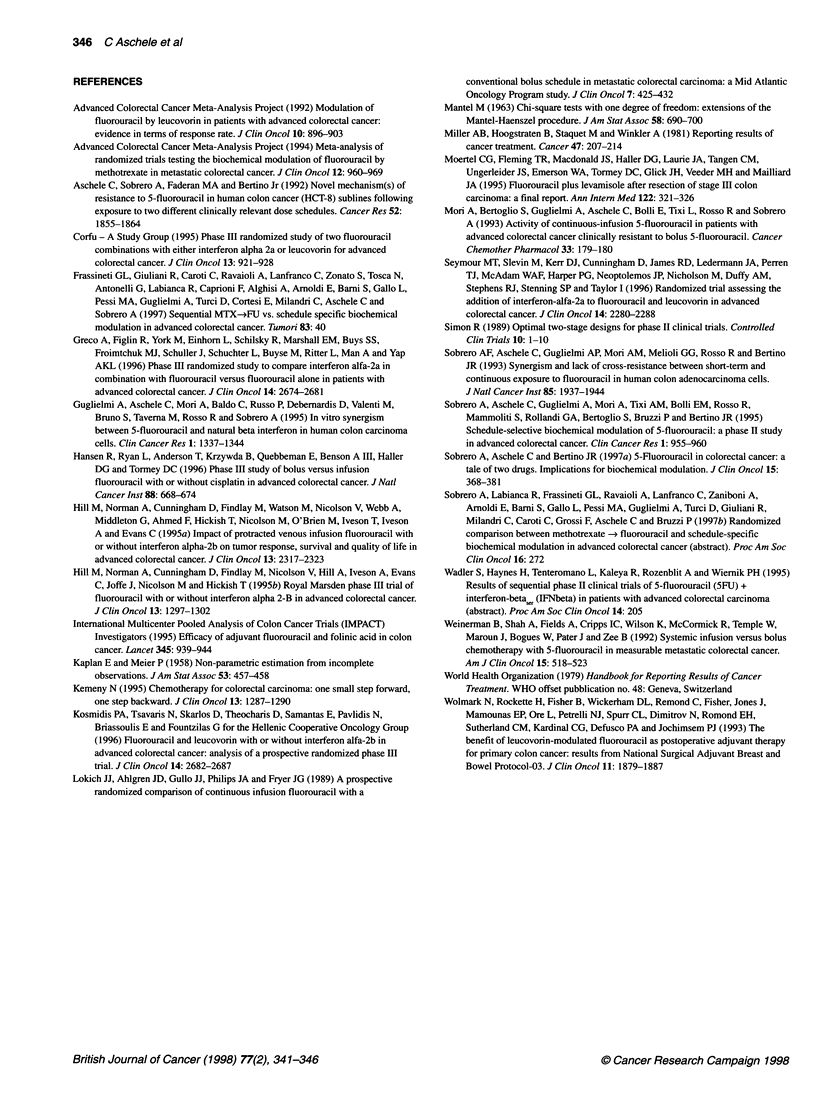# Schedule-selective biochemical modulation of 5-fluorouracil in advanced colorectal cancer: a multicentric phase II study.

**DOI:** 10.1038/bjc.1998.53

**Published:** 1998

**Authors:** C. Aschele, A. Guglielmi, G. L. Frassineti, C. Milandri, D. Amadori, R. Labianca, M. Vinci, L. Tixi, C. Caroti, E. Ciferri, E. Verdi, R. Rosso, A. Sobrero

**Affiliations:** Department of Medical Oncology, Istituto Nazionale per la Ricerca sul Cancro, Genoa, Italy.

## Abstract

We have recently reported high clinical activity against advanced colorectal cancer of a regimen-alternating bolus FUra, modulated by methotrexate (MTX), and continuous infusion FUra, modulated by 6-s-leucovorin (6-s-LV). Considering the low toxicity of the bolus part of this regimen and our recent in vitro finding of a strong synergism between bolus FUra and natural-beta-IFN (n-beta-IFN), this cytokine was incorporated in the bolus part of our treatment programme. Fifty-six patients with untreated, advanced, measurable colorectal cancer were treated with two biweekly cycles of FUra bolus (600 mg m(-2)), modulated by MTX (24 h earlier, 200 mg m(-2)), and n-beta-IFN (3 x 10(6) IU i.m. every 12 h, starting at the time of FUra administration for four doses), alternating with a 3-week continuous infusion of FUra (200 mg m(-2) daily), modulated by 6-s-LV (20 mg m(-2) weekly bolus). After a 1-week rest, the whole cycle (8 weeks) was repeated if indicated. A total of 5 complete and 17 partial responses were obtained (response rate, 41%; 95% confidence limits, 28-55%) in 54 assessable patients. After a median follow-up time of 36 months, five patients are still alive. Overall, the median time to treatment failure was 6.4 months. The median duration of survival was 15.0 months. There was one treatment-related death after a course of MTX --> bolus FUra/n-beta-IFN and grade III-IV toxicity occurred in 18% of the patients. As the addition of n-beta-IFN results in high toxicity, whereas the efficacy seems to be similar to that of the same regimen without the cytokine, our groups are currently randomizing the original regimen, without IFN, against standard modulated bolus FUra.


British Journal of Cancer (1998) 77(2), 341–346
© 1998 Cancer Research Campaign

### Schedule-selective biochemical modulation of 5-fluorouracil in advanced colorectal cancer: a multicentric phase II study

**C Aschele[1], A Guglielmi[1], GL Frassineti[2], C Milandri[3], D Amadori[2], R Labianca[4], M Vinci[4], L Tixi[1], C Caroti[5], E Ciferri[6], E Verdi[1], R Rosso[1] and A Sobrero[7]**

[1]Department of Medical Oncology, Istituto Nazionale per la Ricerca sul Cancro, 16132 Genoa, Italy; [2]Medical Oncology Division, Ospedale Morgagni-Pierantoni, 47100 Forlì, Italy; [3]Istituto Oncologico Romagnolo, 47100 Forlì, Italy; [4]Medical Oncology Division, Ospedale S. Carlo Borromeo, 20153 Milan, Italy; [5]Medical Oncology, Ospedali Galliera, 16128 Genoa, Italy; [6]General Surgery Division, Ospedali S. Martino, 16132 Genoa, Italy; [7]Department of Oncology, University of Genoa, Italy

**Summary** We have recently reported high clinical activity against advanced colorectal cancer of a regimen-alternating bolus FUra, modulated by methotrexate (MTX), and continuous infusion FUra, modulated by 6-s-leucovorin (6-s-LV). Considering the low toxicity of the bolus part of this regimen and our recent in vitro finding of a strong synergism between bolus FUra and natural-β-IFN (n-β-IFN), this cytokine was incorporated in the bolus part of our treatment programme. Fifty-six patients with untreated, advanced, measurable colorectal cancer were treated with two biweekly cycles of FUra bolus (600 mg $m^{-2}$), modulated by MTX (24 h earlier, 200 mg $m^{-2}$), and n-β-IFN ($3 \times 10^6$ IU i.m. every 12 h, starting at the time of FUra administration for four doses), alternating with a 3-week continuous infusion of FUra (200 mg $m^{-2}$ daily), modulated by 6-s-LV (20 mg $m^{-2}$ weekly bolus). After a 1-week rest, the whole cycle (8 weeks) was repeated if indicated. A total of 5 complete and 17 partial responses were obtained (response rate, 41%; 95% confidence limits, 28–55%) in 54 assessable patients. After a median follow-up time of 36 months, five patients are still alive. Overall, the median time to treatment failure was 6.4 months. The median duration of survival was 15.0 months. There was one treatment-related death after a course of MTX → bolus FUra/n-β-IFN and grade III–IV toxicity occurred in 18% of the patients. As the addition of n-β-IFN results in high toxicity, whereas the efficacy seems to be similar to that of the same regimen without the cytokine, our groups are currently randomizing the original regimen, without IFN, against standard modulated bolus FUra.

**Keywords:** advanced colorectal cancer; biochemical modulation; 5-fluorouracil; natural-β-interferon; schedule of administration

A substantial improvement has been achieved in the adjuvant treatment of colorectal cancer in recent years (Moertel et al, 1989; Wolmark et al, 1993; IMPACT investigators, 1995), whereas only marginal progress have been made in the advanced stage (Kemeny, 1995).

As shown in two recent meta-analysis articles (Advanced Colorectal Cancer Meta-Analysis Project, 1992; Advanced Colorectal Cancer Meta-Analysis Project, 1994), the addition of either leucovorin (LV) or methotrexate (MTX) to bolus FUra regimens resulted in a doubling of the response rate compared with FUra alone. This enhanced activity was not paralleled by a difference in overall survival with FUra + LV compared with FU alone. The small, although significant, survival advantage for MTX/FUra compared with FUra alone confirms the limits of these biochemically modulated bolus FUra regimens.

Long-term administration of FUra is another rational approach to improve the activity of this agent and enhanced activity compared with bolus administration has been shown in several randomized studies (Lokich et al, 1989; Weinerman et al, 1992; Hansen et al, 1996). However, even this approach failed to result in a survival benefit.

A series of clinical and experimental studies support the hypothesis that FUra has different mechanisms of action depending on the dose schedule (Aschele et al, 1992; Mori et al, 1993; Sobrero et al, 1993). Biochemical modulators specific for each schedule should be used. According to our preclinical data, maximal enhancement of bolus FUra is more probably obtained with drugs that enhance the RNA effect of the fluoropyrimidine, such as MTX, trimetrexate, phosphonacetyl-L-aspartate, 6-MMPR, whereas LV, which enhances the thymidylate synthase (TS) inhibitory activity of FUra, may result in greater potentiation when the fluoropyrimidine is administered as a continuous infusion (Sobrero et al, 1997*a*).

This approach was tested in a phase II clinical study at the Istituto Nazionale per la Ricerca sul Cancro of Genoa. A regimen alternating two biweekly cycles of bolus FUra, preceded by MTX, with a 3-week continuous infusion of FUra, modulated by low dose LV, resulted in a 48% objective response rate, 9.5 months progression free survival (PFS) and 20.2 months overall survival on a series of 33 advanced colon cancer patients (Sobrero et al, 1995).

The low toxicity of the bolus part of this regimen along with our recent in vitro finding of selective potentiation of pulse FUra by n-β-IFN (Guglielmi et al, 1995), prompted us to incorporate the cytokine in the bolus part of our programme to maximize the clinical activity. The dose and timing of IFN administration were chosen on the basis of our in vitro data: exposure of human colon

*Received 19 March 1997*
*Revised 19 June 1997*
*Accepted 24 July 1997*

*Correspondence to:* A Sobrero, Medical Oncology, Istituto Nazionale per la Ricerca sul Cancro, P. Le Rosanna Benzi, 10-16132 Genova, Italy cancer cells (HCT-8) to low-dose n-β-IFN for up to 24 h after a short-term exposure to FUra results in enhanced incorporation of the fluoropyrimidine into nucleic acids and enhanced cell kill (Guglielmi et al, 1995). Natural-β-IFN was thus given for only 2 days after bolus FUra administration and a low dose was used ($3 \times 10^6$ IU twice daily). This leaves the infusional part of the original regimen unchanged and only four i.m. IFN administrations are added to each course of MTX/bolus FUra. A further objective of this clinical trial was to define the activity of schedule-dependent biochemical modulation in a multi-institution setting, before a randomized comparison with standard modulated bolus FUra.

### MATERIALS AND METHODS

### Eligibility criteria

Fifty-six patients with biopsy-proven, relapsed or metastatic adenocarcinoma of the colon or rectum, referred to the Istituto Nazionale per la Ricerca sul Cancro (Genoa, Italy), the Istituto Oncologico Romagnolo (Forlì, Italy) or the Ospedale S. Carlo Borromeo (Milan, Italy) were accrued into this three institution phase II trial from October 1993 to December 1994. The disease had to be measurable, with appropriate radiological examinations obtained no longer than 1 month before the beginning of treatment. No previous chemotherapy for metastatic disease was allowed and adjuvant chemotherapy should have been completed more than 1 year before study entry. Radiation therapy was allowed, as long as it did not encompass the indicator lesions. Adequate bone marrow (granulocyte and platelet counts greater than 1500 $mm^{-3}$ and 100 000 $mm^{-3}$ respectively), hepatic (serum bilirubin $\leq$ 3.0 mg $dl^{-1}$, aspartate and alanine aminotransferases less than three times the upper limits of normal) and renal (creatinine levels $\leq$ 1.7 mg $dl^{-1}$) function was required. ECOG performance status had to be $\leq$ 2 and life expectancy greater than 3 months. Additional eligibility criteria included geographic accessibility, the absence of clinically relevant ascites and the absence of other medical conditions clearly contraindicating the delivery of any chemotherapy.

This study was approved by the ethics committee of the Istituto Nazionale per la Ricerca sul Cancro of Genoa and informed consent was obtained before study entry.

### TREATMENT PLAN

Sequential methotrexate → bolus FUra + n-β-IFN were used alternately with prolonged continuous infusion (CI) FUra modulated by 6-S-LV. Figure 1 illustrates the regimen. The bolus part of the treatment programme consisted of two biweekly administrations of bolus FUra given i.v. at 600 mg $m^{-2}$ (day 2 and 16), modulated by MTX, given 24 h earlier at 200 mg $m^{-2}$ (day 1 and 15), and n-β-IFN, administered i.m. at $3 \times 10^6$ IU every 12 h × 4, starting at the time of FUra administration (days 2–3 and 16–17). 6-S-LV, 10 mg $m^{-2}$ p.o. every 6 h × 6, was given on days 2 and 16, starting after bolus FUra administration, as a rescue from MTX toxicity. To prevent or attenuate the severity of flu-like syndrome, the patients were instructed to take acetaminophen, 500 mg p.o., 30 min before each IFN administration. After a 2-week rest, a 3-week prolonged infusion of FUra at 200 mg $m^{-2}$ $day^{-1}$ was started (day 29–50), modulated by weekly boluses of 6-S-LV at 20 mg $m^{-2}$ (day 29, 36 and 43). The cycles were repeated after 1 week of rest (day 57), provided that the patient had recovered from toxicity. The entire duration of one cycle is thus 8 weeks. CI FUra was administered through an implanted catheter and a venous Port-a-cath (Pharmacia) connected to a portable programmable external pump (CADD-1, Pharmacia).

Toxicity was evaluated according to World Health Organization (WHO) criteria (World Health Organization, 1979) on days 15, 29, 36, 43, 50 and 57. Complete blood counts were obtained on the same days. Liver function tests, blood urea nitrogen, creatinine and electrolytes were obtained monthly.

Dose modification criteria for the MTX → FUra + IFN regimen were as follows: no dose reduction for gastrointestinal grade I and II toxicity; for grade III diarrhoea or mucositis, the treatment was delayed until recovery and the doses of MTX and FUra of the next cycle were decreased by 50%; the dose was reduced by 50% for a WBC of < 3000 $mm^{-3}$ or platelets < 75 000 $mm^{-3}$ on the day of recycling; treatment was discontinued in case of grade IV toxicity; the dose of IFN was not reduced for myelotoxicity, diarrhoea or mucositis unless toxicity was not overcome by reducing the MTX and FUra doses; the dose of IFN was reduced by 50% for severe constitutional symptoms (fatigue, malaise and anorexia).

CI FUra was discontinued at the first signs of mucositis and/or palmar–plantar dysaesthesia/burning, and resumed when these symptoms abated. In the case of severe (grade III) mucositis, the infusion was resumed at a reduced FUra dose (50%). The dose of LV during the infusional treatment was not modified in this study.

**Table 1** Patient characteristics ($n$ = 56)

| Characteristic | $n$ (%) |
|---|---|
| Age, years | |
| Median | 63 |
| Range | 43–83 |
| Male | 32 (56) |
| Female | 24 (44) |
| ECOG PS | |
| 0 | 29 (52) |
| 1 | 22 (39) |
| 2 | 5 (9) |
| Site of primary | |
| Colon | 43 (77) |
| Rectum | 13 (23) |
| Previous adjuvant chemotherapy | 10 (18) |
| Sites of disease | |
| Liver only | 33 (59) |
| Liver and other sites | 10 (18) |
| Lung | 3 (5) |
| Intra-abdominal | 10 (18) |

### Response evaluation

Patients who had received at least 2 months of therapy (one cycle) with adequate pretreatment and follow-up radiographic studies were considered assessable for response, as were patients who experienced rapid disease progression after at least two courses of bolus FUra.

Objective responses were evaluated according to WHO criteria (Miller et al, 1981) after each cycle of treatment (2 months); the baseline areas of the indicator lesions ($cm^2$) and their variations at each successive cycle were reported.

© Cancer Research Campaign 1998

**Figure 1** Design of drug regimen. One cycle = 8 weeks. In the first part of the cycle, patients were given MTX 200 mg $m^{-2}$ i.v. diluted in 500 ml of $D_5W$, infused in 1 h, day 1; FUra 600 mg $m^{-2}$ i.v. bolus, day 2; (6S)LV, 10 mg $m^{-2}$ p.o. every 6 h x 6, days 2–3, starting after FUra bolus; and n-β-IFN 3 x $10^6$ IU i.m. every 12 h x 4, days 2–3. In the second part of the cycle, patients were given FUra, 200 mg $m^{-2}$, day 1 CI x 3 weeks, and (6S)LV, 20 mg $m^{-2}$ i.v. bolus every week

**Figure 2** Kaplan–Meier TTF curve for all 56 patients

**Figure 3** Kaplan–Meier survival curve for all 56 patients. Fifty-one patients have died

### Dose intensity

Delivered dose intensity for bolus FUra and for CI FUra was expressed in terms of mg $m^{-2}$ per week.

### Statistical methods

A 48% response rate was obtained in the previous phase II trial of our regimen with 95% confidence limits at 31 and 66% (Sobrero et al, 1995). If the addition of IFN increases this activity, an interesting target level for the response rate is 60%. The study was thus planned to have a less than 10% probability (β error = 0.10) of rejecting the regimen if the true response rate is at least 60% (P1) and a less than 5% probability (α error = 0.05) of accepting the regimen for further studies if the true response rate is less than 40% (P0).

A two-stage study was planned according to Simon's minimax design (Simon et al, 1989). More than 12 responses had to occur among the first 29 patients (I stage) to proceed to the second stage. At the end of the study (54 patients), more than 27 responses were required to consider the regimen including n-β-IFN for additional studies, whereas IFN had to be dropped if 27 responses or less were seen. The time to treatment failure (TTF) was measured from the initiation of therapy until the date of disease progression as defined above or discontinuation of therapy for toxicity or refusal. The probability of treatment failure and survival was calculated using the Kaplan–Meier method (Kaplan and Meier, 1958). The association between performance status and the proportion of responses was assessed using the Mantel test for trend (Mantel, 1963).

### RESULTS

### Patient characteristics

Between October 1993 and December 1994, 56 patients meeting the eligibility criteria were registered at the three participating Institutions.

Table 1 shows patient characteristics. Ten (18%) patients had received previous adjuvant chemotherapy: four had received FUra-LV, 5 FUra-levamisole and 1 FUra-LV-levamisole.

Thirty-three (59%) patients had liver metastases only, whereas ten (18%) had liver disease plus other sites of metastases. Among the patients without liver involvement, three had lung metastases and ten had extrahepatic intra-abdominal masses as measurable sites.

The median time between diagnosis of metastatic disease and study entry was 40 (range 5–237) days.

Lesions were measured by CT scan in 43 patients and ultrasound in seven patients the remainder being measured by chest radiography and nuclear magnetic resonance. Only eight patients had lesions less than 2 x 2 cm and the median measured baseline tumour area was 35 (range 2–358) $cm^2$. The median number of tumour lesions evaluated per patient was three (range 1–7) and eight patients had only one lesion measured.

All patients were assessable for toxicity. Fifty-four patients were considered assessable for response. One patient was excluded from response assessment because measurements were not available for the tumour lesions detected in the baseline liver ultrasound. The other patient had enlarged supraclavicular lymph nodes as the only site of metastatic disease. Measurements were obtained by physical examination only, rather than radiographic imaging as specified by the protocol. This patient was therefore not included in the response analysis, despite a reduction in tumour mass assessed by physical examination and a greater than 50% reduction in CEA levels. The time to disease progression in this patient was 8 months and he is still alive at 27 months. All patients were included in the analysis of TTF and survival.

**Table 2** Clinical toxicity: worst WHO grade per patient across all cycles ($n$ = 56 patients)

| | MTX → FUra + β-IFN toxicity grade (%) | | | | CI FUra + 6-S-LV toxicity grade (%) | | | |
|---|---|---|---|---|---|---|---|---|
| **Toxicity** | **I** | **II** | **III** | **IV** | **I** | **II** | **III** | **IV** |
| Mucositis | 27 | 9 | 7 | 2 | 21 | 34 | 9 | – |
| Diarrhoea | 25 | 4 | 4 | – | 14 | 11 | 9 | – |
| Nausea/vomiting | 53 | 12 | – | – | 30 | 7 | – | – |
| Asthenia | 21 | 20 | 2 | – | 11 | 5 | 2 | – |
| Fever/myalgia | 18 | 37 | 2 | – | 9 | 4 | – | – |
| Anaemia | 16 | 7 | – | – | 4 | 4 | – | – |
| Thrombocytopenia | – | – | 2 | 4 | 2 | – | – | – |
| Leucopenia | 7 | 5 | 2 | 5 | 2 | – | – | – |
| Conjunctivitis[a] | 32 | – | – | – | 28 | – | – | – |
| Hand–foot syndrome[a] | 12 | – | – | – | 30 | – | – | – |

[a]Scored as grade 1 if present.

### Treatment outcome

Five complete responses (CRs) and 17 PRs were observed among the 54 patients considered assessable for response, for an overall response rate of 41% (95% confidence interval, 28–55%). If all the 56 patients are included, the response rate was 39%. In addition, a substantial percentage of patients (39%) had a minor response or stable disease with a median duration of 4.5 (range 2.0–9.2) months. Eleven failures were reported: five patients progressed after the first cycle of treatment, three patients showed a rapid disease progression before the end of the first cycle and three patients had the treatment interrupted after the first two courses of bolus FUra because of grade IV toxicity.

The median time to achieve a partial or complete response was 58 (range, 49–220) days, with initial responses attained after one cycle (ten cases), two cycles (seven cases) and three cycles (five cases). Half of the responding patients showed continued tumour shrinkage and the median time to achieve the maximum clinical response was 130 (range 54–287) days.

Four out of five patients with complete response had liver disease only, with multiple inoperable metastases (two, three, three and six measured lesions respectively); the other patient had three lung lesions as the only site of metastatic disease. Two of the patients with liver disease underwent surgical exploration 2 and 6 months after achieving the complete response respectively. No residual disease was found in the first patient who is still alive and disease-free at 40 months. A peritoneal dissemination (multiple unresectable peritoneal nodules) was revealed in the second patient who had failed at this point (CR duration 6 months) and died 5 months later. The other complete responses lasted 8, 9 and 13 months.

Only 4 out of the 22 responses were obtained in patients with 2 metastatic sites (liver + pelvic masses), the rest being liver only (15 patients), lung only (one patient) and extrahepatic intra-abdominal disease (two patients).

Previous adjuvant treatment appeared to influence the clinical response: one out of ten patients who had received adjuvant treatment responded (10% response rate), whereas 21 responses were observed among the 44 patients who had not received previous adjuvant chemotherapy (48% response rate, $P = 0.038$).

The combined CR and partial response (PR) rate was 54%, 29% and 20% in patients with an ECOG PS of 0,1 and 2, respectively ($\chi^2 = 3.755$, $P = 0.052$, two-tailed Mantel test for trend), suggesting that initial PS affects treatment outcome as reported earlier.

Age and primary site did not appear to influence the overall clinical response (47% and 37% in patients younger and older than 60 years of age; 42% and 36% in patients with colon or rectal primaries).

The median duration of response was 6.8 (range, 2–40+) months.

All patients are now off treatment. Three patients declined further chemotherapy while they were still responding; they were considered treatment failures as of the date the treatment was discontinued. The patient that achieved a pathological CR is still disease-free; all the rest have progressed. With a median follow-up time of 36 months, 51 deaths have occurred. The median TTF (Figure 2) for the whole cohort of 56 patients was 6 (range 0–40) months and the median survival time (Figure 3) was 15.0 (range 0–40) months.

### Dose delivery and toxicity

One-hundred and fifty-six cycles of treatment (2 months each) were administered, with a median of three cycles (range 0–5) per patient. The number of 'bolus' FUra administrations (309) is consistent with the total number of cycles administered. The number of weeks of CI FUra (410) is slightly lower than expected. Eighteen cycles consisted of less than 3 weeks of CI FUra because of toxicity (11 cycles), catheter-related complications (four cycles) and patient refusal (three cycles). In addition, progression was documented and treatment discontinued before or during the infusional part of the regimen in ten cycles.

Ten (3.2%) of 309 'bolus FUra' administrations were given at reduced doses because of toxicity related to the previous courses. Thirty-five (8.5%) of 410 weeks of CI FUra were administered for less than 7 days and/or at reduced doses because of toxicity (27 weeks) or other reasons including catheter-related complications, disease progression and patient request (8 weeks).

It has been reported that FUra dose intensity may affect treatment outcome. In the current study, no substantial differences

 © Cancer Research Campaign 1998

between responders and non-responders were detected in the median FUra dose intensity (mg $m^{-2}$ per week) actually delivered for the first two cycles of treatment: 271 (range 103–311) vs 278 (range 190–295) for the 'bolus part' ($n$ = 43) and 848 (range 350–1131) vs 835 (range 126–1025) for the CI part ($n$ = 40). These figures did not change substantially throughout the treatment. The median delivered FUra dose intensity over all cycles of therapy was 289 mg $m^{-2}$ per week for the 'bolus part' (range 103–320) and 924 mg $m^{-2}$ per week for the CI part (range 135–1131). These values represent 96.3% and 88% of planned dose intensity values respectively.

Table 2 reports the worst toxicity of each type, suffered by each patient, across all cycles. The two parts of the regimen are considered separately. One toxic death was reported after a course of bolus FU modulated with MTX and β-IFN (grade IV leucopenia, thrombocytopenia and mucositis with septic shock). In this part of the programme, severe or life-threatening toxicity occurred in 14% of the patients (leucopenia, mucositis and diarrhoea). Grade IV toxicity was not reported during the infusional part of the regimen. A total of 18% of the patients experienced grade III mucositis and/or diarrhoea during the administration of CI FUra. A total of 39% of the patients experienced mild to moderate flu-like syndrome in the bolus part of the programme, leading to a reduction of the n-β-IFN dose in three patients. Hand–foot syndrome was much more prominent during the infusional treatment (30% of patients), whereas conjunctivitis was observed only after several months of treatment and could not be attributed to either of the two schedules. Only three patients (5%) had catheter-related complications requiring admission to hospital: one with sepsis and two with thrombosis.

### DISCUSSION

The use of biochemical modulators specific for each schedule of FUra administration along with the alternate use of bolus and CI FUra represents a novel strategy to improve the efficacy of the fluoropyrimidine. This approach resulted in high clinical activity in a recent phase II trial of a regimen alternating bolus FUra, modulated by MTX, and CI FUra, modulated by low-dose 6-s-LV (Sobrero et al, 1995).

This strategy is based on a series of experimental and clinical findings: (a) repeated short-term exposures of human colon cancer cells to FUra in vitro produced resistance via an RNA-related mechanism, whereas resistance to long-term exposures was mediated by a TS-directed mechanism (Aschele et al, 1992); (b) in the same in vitro model, cells resistant to pulse FUra maintained sensitivity to prolonged exposures to the fluoropyrimidine (Sobrero et al, 1993); and (c) patients progressing during treatment with bolus FUra were not completely resistant to CI FUra (Mori et al, 1993). These findings suggested that FUra may have two different modes of action depending on the schedule of administration. Bolus and CI FUra may thus be alternated to prevent or delay the development of drug resistance and it may be possible to selectively modulate each schedule biochemically. According to our preclinical data, potentiation of FUra by LV may be maximal when the fluoropyrimidine is given for prolonged periods of time whereas channelling FUra into RNA using MTX may improve results when a short-term, high dose is used (Sobrero et al, 1997*b*).

The present study was designed to test whether the addition of n-β-IFN could further enhance the activity of the original regimen. Natural β IFN was used on the basis of experimental data showing synergistic interactions with FUra on human colon cancer cells in vitro (Guglielmi et al, 1995). In this experimental system, n-β-IFN had stronger cytotoxic effects compared with α-IFN (Guglielmi et al, 1995). In addition, six randomized studies failed to demonstrate a significant improvement in response rate or survival in patients treated with FUra (± LV) + α-IFN compared with bolus FUra alone, CI FUra alone or bolus FUra + LV (Corfu – A Study Group, 1995; Hill et al, 1995*a, b*; Greco et al, 1996; Kosmidis et al, 1996; Seymour et al 1996). Preliminary clinical data had been reported on the use of β-IFN as FUra modulator: substantial clinical activity and low toxicity were obtained in a series of clinical trials on advanced colorectal cancer patients (Wadler et al, 1995).

Our in vitro data also provided the rationale for the selective addition of the cytokine in the bolus part of the original regimen as well as for the timing and duration of IFN administration. The synergism obtained in vitro was strictly dependent on FUra scheduling with long-term exposures to the fluoropyrimidine resulting in loss of the synergistic interactions observed with short-term exposures (Guglielmi et al, 1995). Biochemical studies indicated that exposure to low-dose IFN for up to 24 h after a short-term exposure to FUra resulted in enhanced incorporation of the fluoropyrimidine into nucleic acids (Guglielmi et al, 1995). On this basis, IFN was only given for 2 days after bolus FUra administration and a low dose was used ($3 \times 10^6$ IU twice daily).

Despite the sound preclinical rationale, the addition of n-β-IFN does not appear to improve the therapeutic outcome compared with the original regimen. The response rate, median TTF and median overall survival in the current study are slightly lower than our previous phase II study (Sobrero et al, 1995). As this was not a randomized comparison, a different distribution of prognostic factors affecting the clinical response to FUra between the patient populations of the two studies may contribute to explain these results. In the study reported here, four patients were found to have brain metastases leading to treatment discontinuation within the first cycle, while brain involvement was a later event in the previous trial. In addition, the percentage of patients that previously received adjuvant therapy is double that in our previous study. However, the main patient characteristics, including median age and PS, the percentage of patients asymptomatic or minimally symptomatic, the measured baseline tumour area and the sites of measurable disease, were similar between the two studies. It is thus reasonable to infer from the results of this trial that further selective anti-tumour modulation of bolus FUra by n-β-IFN is not obtained at the clinical level.

Even although the addition of IFN does not seem to improve the outcome of our regimen, the results reported here show a substantial activity of schedule-oriented biochemical modulation. The 15 months in median survival time along with a 41% response rate in 56 patients accrued at three different institutions, compares well with the best reported results of modulated FUra regimens. The durability of CRs and PRs is also promising. Our group has thus discarded n-β-IFN but has maintained the original alternating regimen as the experimental arm of a currently ongoing randomized trial comparing schedule selective biochemical modulation vs standard modulated bolus FUra. Preliminary response data (34% vs. 12%, $P = 0.001$) and TTF (6.2 months vs. 4.1 months, $P = 0.01$) are extremely promising (Sobrero et al, 1997*b*; Frassineti et al, 1997).

### AKNOWLEDGEMENTS

This study was supported by grants CNR ACRO 95.00447.PF39, AIRC 1996, AIRC 1997, CNR BTBS 93.01119.PF70.

© Cancer Research Campaign 1998 

### REFERENCES

Advanced Colorectal Cancer Meta-Analysis Project (1992) Modulation of fluorouracil by leucovorin in patients with advanced colorectal cancer: evidence in terms of response rate. *J Clin Oncol* **10**: 896–903

Advanced Colorectal Cancer Meta-Analysis Project (1994) Meta-analysis of randomized trials testing the biochemical modulation of fluorouracil by methotrexate in metastatic colorectal cancer. *J Clin Oncol* **12**: 960–969

Aschele C, Sobrero A, Faderan MA and Bertino Jr (1992) Novel mechanism(s) of resistance to 5-fluorouracil in human colon cancer (HCT-8) sublines following exposure to two different clinically relevant dose schedules. *Cancer Res* **52**: 1855–1864

Corfu – A Study Group (1995) Phase III randomized study of two fluorouracil combinations with either interferon alpha 2a or leucovorin for advanced colorectal cancer. *J Clin Oncol* **13**: 921–928

Frassineti GL, Giuliani R, Caroti C, Ravaioli A, Lanfranco C, Zonato S, Tosca N, Antonelli G, Labianca R, Caprioni F, Alghisi A, Arnoldi E, Barni S, Gallo L, Pessi MA, Guglielmi A, Turci D, Cortesi E, Milandri C, Aschele C and Sobrero A (1997) Sequential MTX→FU vs. schedule specific biochemical modulation in advanced colorectal cancer. *Tumori* **83**: 40

Greco A, Figlin R, York M, Einhorn L, Schilsky R, Marshall EM, Buys SS, Froimtchuk MJ, Schuller J, Schuchter L, Buyse M, Ritter L, Man A and Yap AKL (1996) Phase III randomized study to compare interferon alfa-2a in combination with fluorouracil versus fluorouracil alone in patients with advanced colorectal cancer. *J Clin Oncol* **14**: 2674–2681

Guglielmi A, Aschele C, Mori A, Baldo C, Russo P, Debernardis D, Valenti M, Bruno S, Taverna M, Rosso R and Sobrero A (1995) In vitro synergism between 5-fluorouracil and natural beta interferon in human colon carcinoma cells. *Clin Cancer Res* **1**: 1337–1344

Hansen R, Ryan L, Anderson T, Krzywda B, Quebbeman E, Benson A III, Haller DG and Tormey DC (1996) Phase III study of bolus versus infusion fluorouracil with or without cisplatin in advanced colorectal cancer. *J Natl Cancer Inst* **88**: 668–674

Hill M, Norman A, Cunningham D, Findlay M, Watson M, Nicolson V, Webb A, Middleton G, Ahmed F, Hickish T, Nicolson M, O'Brien M, Iveson T, Iveson A and Evans C (1995*a*) Impact of protracted venous infusion fluorouracil with or without interferon alpha-2b on tumor response, survival and quality of life in advanced colorectal cancer. *J Clin Oncol* **13**: 2317–2323

Hill M, Norman A, Cunningham D, Findlay M, Nicolson V, Hill A, Iveson A, Evans C, Joffe J, Nicolson M and Hickish T (1995*b*) Royal Marsden phase III trial of fluorouracil with or without interferon alpha 2-B in advanced colorectal cancer. *J Clin Oncol* **13**: 1297–1302

International Multicenter Pooled Analysis of Colon Cancer Trials (IMPACT) Investigators (1995) Efficacy of adjuvant fluorouracil and folinic acid in colon cancer. *Lancet* **345**: 939–944

Kaplan E and Meier P (1958) Non-parametric estimation from incomplete observations. *J Am Stat Assoc* **53**: 457–458

Kemeny N (1995) Chemotherapy for colorectal carcinoma: one small step forward, one step backward. *J Clin Oncol* **13**: 1287–1290

Kosmidis PA, Tsavaris N, Skarlos D, Theocharis D, Samantas E, Pavlidis N, Briassoulis E and Fountzilas G for the Hellenic Cooperative Oncology Group (1996) Fluorouracil and leucovorin with or without interferon alfa-2b in advanced colorectal cancer: analysis of a prospective randomized phase III trial. *J Clin Oncol* **14**: 2682–2687

Lokich JJ, Ahlgren JD, Gullo JJ, Philips JA and Fryer JG (1989) A prospective randomized comparison of continuous infusion fluorouracil with a conventional bolus schedule in metastatic colorectal carcinoma: a Mid Atlantic Oncology Program study. *J Clin Oncol* **7**: 425–432

Mantel M (1963) Chi-square tests with one degree of freedom: extensions of the Mantel-Haenszel procedure. *J Am Stat Assoc* **58**: 690–700

Miller AB, Hoogstraten B, Staquet M and Winkler A (1981) Reporting results of cancer treatment. *Cancer* **47**: 207–214

Moertel CG, Fleming TR, Macdonald JS, Haller DG, Laurie JA, Tangen CM, Ungerleider JS, Emerson WA, Tormey DC, Glick JH, Veeder MH and Mailliard JA (1995) Fluorouracil plus levamisole after resection of stage III colon carcinoma: a final report. *Ann Intern Med* **122**: 321–326

Mori A, Bertoglio S, Guglielmi A, Aschele C, Bolli E, Tixi L, Rosso R and Sobrero A (1993) Activity of continuous-infusion 5-fluorouracil in patients with advanced colorectal cancer clinically resistant to bolus 5-fluorouracil. *Cancer Chemother Pharmacol* **33**: 179–180

Seymour MT, Slevin M, Kerr DJ, Cunningham D, James RD, Ledermann JA, Perren TJ, McAdam WAF, Harper PG, Neoptolemos JP, Nicholson M, Duffy AM, Stephens RJ, Stenning SP and Taylor I (1996) Randomized trial assessing the addition of interferon-alfa-2a to fluorouracil and leucovorin in advanced colorectal cancer. *J Clin Oncol* **14**: 2280–2288

Simon R (1989) Optimal two-stage designs for phase II clinical trials. *Controlled Clin Trials* **10**: 1–10

Sobrero AF, Aschele C, Guglielmi AP, Mori AM, Melioli GG, Rosso R and Bertino JR (1993) Synergism and lack of cross-resistance between short-term and continuous exposure to fluorouracil in human colon adenocarcinoma cells. *J Natl Cancer Inst* **85**: 1937–1944

Sobrero A, Aschele C, Guglielmi A, Mori A, Tixi AM, Bolli EM, Rosso R, Mammoliti S, Rollandi GA, Bertoglio S, Bruzzi P and Bertino JR (1995) Schedule-selective biochemical modulation of 5-fluorouracil: a phase II study in advanced colorectal cancer. *Clin Cancer Res* **1**: 955–960

Sobrero A, Aschele C and Bertino JR (1997*a*) 5-Fluorouracil in colorectal cancer: a tale of two drugs. Implications for biochemical modulation. *J Clin Oncol* **15**: 368–381

Sobrero A, Labianca R, Frassineti GL, Ravaioli A, Lanfranco C, Zaniboni A, Arnoldi E, Barni S, Gallo L, Pessi MA, Guglielmi A, Turci D, Giuliani R, Milandri C, Caroti C, Grossi F, Aschele C and Bruzzi P (1997*b*) Randomized comparison between methotrexate → fluorouracil and schedule-specific biochemical modulation in advanced colorectal cancer (abstract). *Proc Am Soc Clin Oncol* **16**: 272

Wadler S, Haynes H, Tenteromano L, Kaleya R, Rozenblit A and Wiernik PH (1995) Results of sequential phase II clinical trials of 5-fluorouracil (5FU) + interferon-beta$_{ser}$ (IFNbeta) in patients with advanced colorectal carcinoma (abstract). *Proc Am Soc Clin Oncol* **14**: 205

Weinerman B, Shah A, Fields A, Cripps IC, Wilson K, McCormick R, Temple W, Maroun J, Bogues W, Pater J and Zee B (1992) Systemic infusion versus bolus chemotherapy with 5-fluorouracil in measurable metastatic colorectal cancer. *Am J Clin Oncol* **15**: 518–523

World Health Organization (1979) *Handbook for Reporting Results of Cancer Treatment*. WHO offset pubblication no. 48: Geneva, Switzerland

Wolmark N, Rockette H, Fisher B, Wickerham DL, Remond C, Fisher, Jones J, Mamounas EP, Ore L, Petrelli NJ, Spurr CL, Dimitrov N, Romond EH, Sutherland CM, Kardinal CG, Defusco PA and Jochimsem PJ (1993) The benefit of leucovorin-modulated fluorouracil as postoperative adjuvant therapy for primary colon cancer: results from National Surgical Adjuvant Breast and Bowel Protocol-03. *J Clin Oncol* **11**: 1879–1887

© Cancer Research Campaign 1998